# Two New Chromone Derivatives from a Marine Algicolous Fungus *Aspergillus versicolor* GXIMD 02518 and Their Osteoclastogenesis Inhibitory Activity

**DOI:** 10.3390/md23110429

**Published:** 2025-11-07

**Authors:** Xin Qi, Zhen Li, Miaoping Lin, Humu Lu, Shuai Peng, Huangxue Qin, Yonghong Liu, Chenghai Gao, Xiaowei Luo

**Affiliations:** 1 Guangxi Key Laboratory of Marine Drugs, University Engineering Research Center of High-Efficient Utilization of Marine Traditional Chinese Medicine Resources, Guangxi, Institute of Marine Drugs, Guangxi University of Chinese Medicine, Nanning 530200, China; 2 School of Basic Medical Sciences, Hunan University of Medicine, Huaihua 418000, China

**Keywords:** marine algae, marine fungi, *Aspergillus versicolor*, chromones, polyketides, osteoclastogenesis

## Abstract

Two new chromone derivatives, cnidimols I and J (**1** and **2**), together with ten known aromatic derivatives (**3**–**12**), were isolated from the Beibu Gulf algicolous fungus *Aspergillus versicolor* GXIMD 02518. Their structures were determined by comprehensive physicochemical and spectroscopic data interpretation. The absolute configurations of **1** and **2** were accomplished by ECD calculations and X-ray diffraction analysis. Compound **1** was obtained as a pair of enantiomers, which were separated by chiral-phase HPLC analysis. Notably, 3,7-dihydroxy-1,9-dimethyldibenzofuran (**6**) displayed significant inhibition in LPS-induced NF-κB luciferase activity in RAW 264.7 macrophages, which further inhibited RANKL-induced osteoclast differentiation without cytotoxicity in bone marrow macrophage cells.

## 1. Introduction

Osteoporosis is a systemic skeletal disorder characterized by reduced bone mass, disrupted bone microarchitecture, increased bone fragility, and a heightened risk of fractures [1,2,3]. With the accelerating aging of the global population, osteoporosis has emerged as a critical public health challenge [4]. Although some exemplary medications are available for treating osteoporosis that target osteoclasts, such as denosumab, bisphosphate zoledronic acid, and estrogen replacement therapy, they also come with serious side effects [5,6]. Osteoclast differentiation is principally regulated by macrophage colony stimulating factor (M-CSF) and receptor activator of nuclear factor-κB ligand (RANKL) [7]. In recent years, the RANKL signaling pathway has been recognized as a core therapeutic target for inhibiting osteoclast differentiation and attenuating excessive bone resorption [8]. Thus, the discovery and development of natural products that specifically target RANKL-induced osteoclast differentiation hold great significance for the safe and effective treatment of osteoporosis.

**Figure 1:**
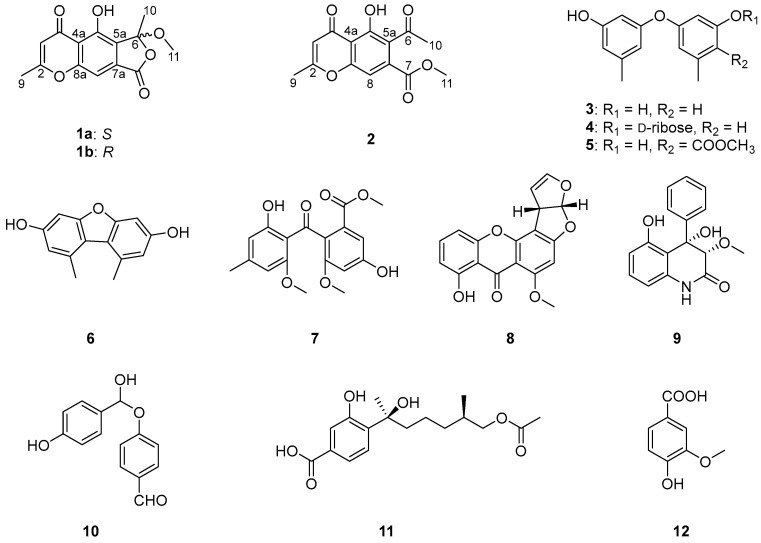
Chemical structures of compounds **1**–**12**.

As a continuing source of novel drug leads, natural products (NPs) have received extensive attention for the treatment of osteoporosis. We have identified a series of inhibitors against osteoclastogenesis with potential for treating osteoporosis from marine fungi, which indicates that it is an important resource for the discovery of novel antiosteoporosis lead compounds [9,10,11,12,13,14]. In this study, two new chromone derivatives, cnidimols I and J (**1** and **2**), together with ten known aromatic derivatives (**3**–**12**), were isolated from the Beibu Gulf algicolous fungus, *Aspergillus versicolor* GXIMD 02518 (Figure 1). Several of them showed inhibitory effects on osteoclastogenesis by inhibiting RANKL-induced NF-kB activation. Herein, their isolation, structure elucidation, and biological activities are described in detail.

## 2. Results and Discussion

**Figure 2:**
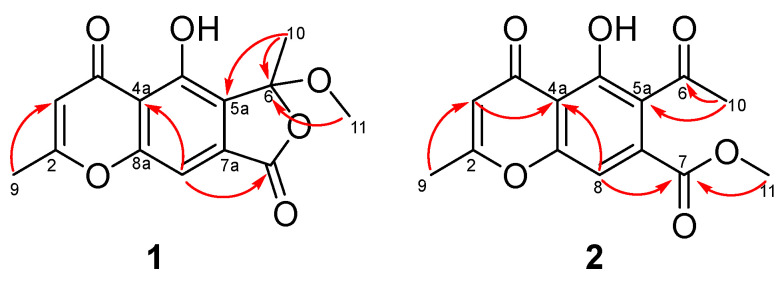
Key HMBC correlations of compounds **1** and **2**.

**Figure 3:**
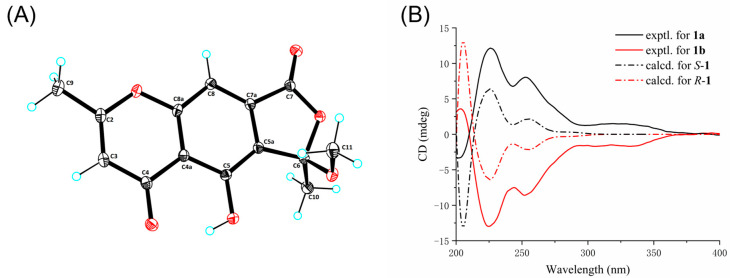
ORTEP drawing of compound **1** (**A**). The experimental and calculated ECD spectra of **1a** and **1b** (**B**).

Cnidimol I (**1**) was obtained as a brown solid, and its molecular formula was determined as C_14_H_12_O_6_ by its HRESIMS data (*m*/*z* 299.0537 [M + Na]^+^, calcd. for C_14_H_12_O_6_Na^+^, 299.0532), suggesting nine degrees of unsaturation. The ^1^H NMR data (Table 1) displayed characteristic signals for two methyls at *δ*_H_ 1.94 (s, H_3_-9) and 2.46 (s, H_3_-10), and one *O*-methyl at *δ*_H_ 3.17 (s, 6-OCH_3_), two olefinic protons at *δ*_H_ 6.23 (s, H-3) and 7.36 (s, H-8). The ^13^C NMR spectrum (Table 1) of **1** exhibited the presence of two methyls (*δ*_C_ 20.9 and 23.8), one methoxy group (*δ*_C_ 51.8), two methines (*δ*_C_ 103.9 and 109.8), and nine nonprotonated carbons (*δ*_C_ 183.9, 169.4, 166.7, 158.3, 156.5, 133.8, 126.2, 113.4, and 109.3). The above NMR data of **1** were similar to those of taeniolin [15], except for obvious differences in ring C. The HMBC correlations of H-8/C-7, H_3_-10/C-5a, C-6, and H_3_-11/C-6 (Figure 2), along with the remaining one degree of unsaturation, allowed the establishment of the furanone ring C in **1**, while both methoxy and methyl groups were located at C-6. Fortunately, the single crystal structure of **1** was achieved after several attempts and analyzed by X-ray diffraction (XRD) method through a Cu K*α* radiation (Figure 3A). The single crystals of **1** had a centrosymmetric space group (P2_1_/c), revealing a racemic mixture, which was also confirmed by nearly no Cotton effect in the experimental ECD spectrum and a barely measurable optical rotation value. Therefore, compounds **1a** and **1b** were subsequently separated using a chiral column of Sepax Chiralomix SA. To determine the absolute configuration of **1**, the experimental and simulated spectra were generated by time-dependent density functional theory (TDDFT) at B3LYP/6−31+G (d). The calculated ECD curves of *S*-**1** and *R*-**1** were well in accord with the experimental curves of **1a** and **1b**, respectively (Figure 3B). Thus, the absolute configurations of **1a** and **1b** were assigned as *S* and *R*, respectively. Compounds **1a** and **1b** were deduced as a pair of chromone enantiomers named as (+)-cnidimol I and (–)-cnidimol I, respectively.

Cnidimol J (**2**) was obtained as a brown solid, and its molecular formula was determined as C_14_H_12_O_6_ by its HRESIMS data (*m*/*z* 299.0531 [M + Na]^+^, calcd. for C_14_H_12_O_6_Na^+^, 299.0532), which suggested nine degrees of unsaturation. Compound **2** was the isomer of **1** according to the HRESIMS data and UV spectrum. The NMR data of **2** was almost identical to those of **1**, suggesting their structural similarity, and the major difference was the ring C. The methyl ester group (*δ*_C_ 167.4) was deduced to be connected at C-7a, evidenced by HMBC correlations from H-8 to C-7 and from H-11 to C-7. The HMBC correlations from H-10 to C-6 and C-5a indicated the carbonyl group (*δ*_C_ 203.1) was located between C-5a and C-10 (Figure 2). Hence, the planar structure of cnidimol J was determined.

According to the literature reports, cnidimols A–H were isolated from the aerial parts and fruits of *Cnidium monnieri* [16,17]. Compounds **1** and **2** shared chromone skeletons with these cnidimol derivatives, which were accordingly named cnidimols I and J, respectively. Compounds **1** and **2** were likely obtained as artificial products of a putative precursor bearing a carboxylic acid at C-7a, since methanol was used in the separation and purification processes. Compound **1** is possibly derived from the precursor by intramolecular nucleophilic addition reaction [18] and followed by methylation. Meanwhile, compound **2** may be directly originated from the precursor by methylation of the carboxylic acid group.

The other ten known compounds were elucidated as diorcinol (**3**) [19], diorcinol-3-*O*-*α*-d-ribofuranoside (**4**) [20], methyl 2-hydroxy-4-(3-hydroxy-5-methylphenoxy)-6-methylbenzoate (**5**) [21], 3,7-dihydroxy-1,9-dimethyldibenzofuran (**6**) [22], monomethylsulochrin (**7**) [23], sterigmatocystin (**8**) [24], aflaquinolone E (**9**) [25], 4-(hydroxy(4-hydroxyphenyl) methoxy) benzaldehyde (**10**) [26], aspergoterpenin D (**11**) [27], and vanillic acid (**12**) [28], respectively, by comparisons of NMR data with the literature data.

**Figure 4:**
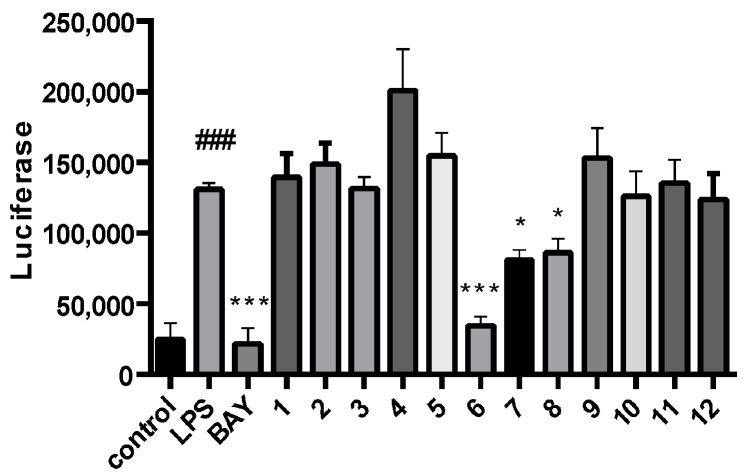
The inhibitory effects of compounds **1**−**12** on LPS-induced NF-κB activation in RAW264.7 cells at 20 μM. *n* = 3. The luciferase activities were measured. ### *p* < 0.001 relative to untreated controls, * *p* < 0.05 and *** *p* < 0.001 compared to LPS-treated controls. BAY (BAY11-7082 treated, positive control).

**Figure 5:**
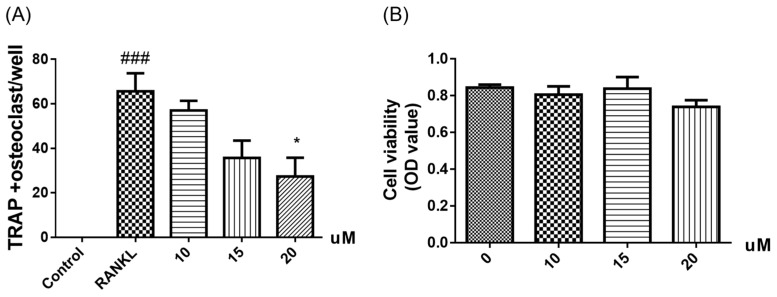
TRAP-positive multinucleated cells were quantified (**A**). Cell viability of compound **6** at different concentrations in BMMs for 72 h was measured by cell counting kit 8 assay (**B**). *n* = 3. ^###^ *p* < 0.001 vs. untreated control, * *p* < 0.05 vs. RANKL-treated control.

**Figure 6:**
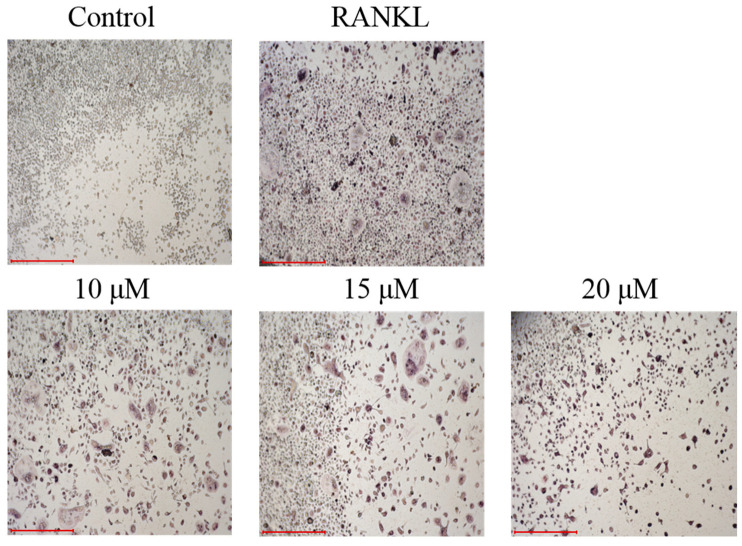
Representative images showing that RANKL-induced osteoclast differentiation was inhibited by compound **6** in a dose-dependent manner in BMMs. (magnification = 100×; scale bar = 500 μm).

**Figure 7:**
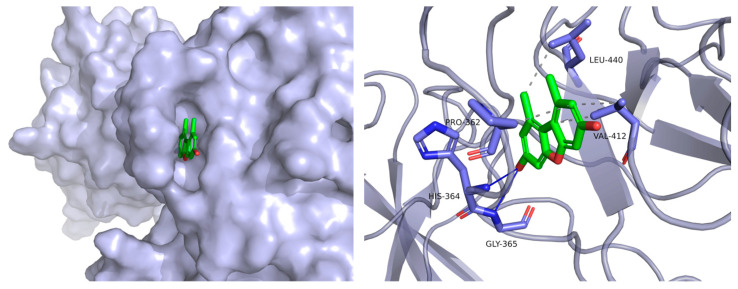
Low-energy binding conformations of **6** bound to NF-κB p65 protein (PDB ID: 3GUT) generated by molecular docking.

All of the compounds were tested for their inhibitory activities of LPS-induced NF-κB activation in RAW264.7 cells. Compounds **6**–**8** exhibited inhibition of lipopolysaccharide (LPS)-induced NF-κB luciferase activities in RAW 264.7 macrophages at 20 µM (Figure 4). Moreover, further investigations were conducted to evaluate their effects on RANKL-induced osteoclast generation by tartrate-resistant acidic phosphatase activity (TRAP) assays. Compound **6** could inhibit RANKL-induced osteoclast differentiation in bone marrow macrophage cells (BMMs) without visible evidence of cytotoxicity (Figure 5 and Figure 6). Subsequently, molecular docking analysis was conducted to gain functional and structural insights between compound **6** and NF-κB p65 protein (PDB ID: 3GUT). The results showed that **6** could interact with NF-κB p65 protein at the entrance of the catalytic pocket, with a calculated binding affinity of −7.4 kcal/mol. As shown in Figure 7, the hydroxy group of **6** formed two hydrogen bonds with the active site residue HIS-364 and GLY-365. Additionally, multiple hydrophobic interactions were also observed in the molecular docking results. This is the first report that the 3,7-dihydroxy-1,9-dimethyldibenzofuran (**6**) could inhibit RANKL-induced osteoclast differentiation through the TRAP assay.

Through a systematic comparison of the structural characteristics of compounds **3**–**6**, this study preliminarily clarified the structure–activity relationship rules of this class of compounds. From the perspective of biological activity assay results, among the target compounds investigated, only **6** demonstrated significant inhibitory activity against NF-κB signaling pathway activation and osteoclast differentiation processes, while no activity response was observed for **3**–**5**. An in-depth analysis of structural differences indicated that the core factor contributing to the activity discrepancy lies in the differences in their parent nuclear skeletons. Specifically, the tricyclic system of **6** constructs a structural pattern with significantly enhanced molecular rigidity and higher spatial conformational stability through the fusion of two benzene rings and a furan ring. In contrast, the parent nuclei of **3**–**5** adopt an open-ring diphenyl ether structure, which exhibits high conformational flexibility and thus cannot form a stable active conformation. Therefore, the structural transformation from the open-ring skeleton of diphenyl ether to the fused-ring skeleton of dibenzofuran represents a key structural modification step for endowing this class of compounds with anti-osteoclastogenic activity.

## 3. Materials and Methods

### 3.1. General Experimental Procedures

OR data were recorded on an IP-digi300/3 (Shanghai InsMark Instrument Technology Corporation, Shanghai, China). ECD spectra were measured on a JASCO J-1500 polarimeter (JASCO Corporation, Tokyo, Japan). The NMR spectra were obtained on a Bruker Avance spectrometer (Bruker BioSpin, Fällanden, Switzerland) operating at 500 MHz for ^1^H NMR, and 125 MHz for ^13^C NMR, using TMS as an internal standard. HR-ESIMS spectra were collected on a Waters Xevo G2-S TOF mass spectrometer (Waters Corporation, Milford, MA, USA). X-ray diffraction intensity data were collected on an XtalLAB PRO single-crystal diffractometer using Cu K*α* radiation (Rigaku, Kyoto, Japan). TLC and column chromatography (CC) were performed on plates precoated with silica gel GF_254_ (10–40 µm) and over silica gel (200–300 mesh) (Qingdao Marine Chemical Factory, Qingdao, China), respectively. All solvents employed were of analytical grade (Shanghai Titan Scientific Co., Ltd., Shanghai, China). Semi-preparative high-performance liquid chromatography (Semi-pre HPLC) was performed on a Shimadzu SCL-10VAP (Shimadzu, Tokyo, Japan), equipped with an ODS column (YMC-pack ODS-A, YMC Co., Ltd., Kyoto, Japan, 10 × 250 mm, 5 µm, 2 mL/min). The artificial sea salt was a commercial product (Guangzhou Haili Aquarium Technology Company, Guangzhou, China).

### 3.2. Fungal Strain and Fermentation

The strain GXIMD 02518 was isolated from a marine alga, *Caulerpa lentillifera*, that was collected from the Coastal Waters of Leizhou Rare Marine Organisms National Nature Reserve, Guangdong province, China, in April 2023. The fungus generated a white, yellow, hair-like colony with an orange color on the back. It was identified as *Aspergillus versicolor* GXIMD 02518 by a sequence analysis of the internal spacer (ITS) region of the rDNA (GenBank accession no. PX393984, 100% similarity to the reported species accession NR_131277.1). A voucher specimen was deposited at the Institute of Marine Drugs, Guangxi University of Chinese Medicine. The strain GXIMD 02518 was cultured on Müller Hinton broth (MB) agar plates (malt extract 15 g, artificial sea salt 10 g, and agar 18 g) at 25 °C for 7 days. Then, it was inoculated in the seed medium (malt extract 15 g and artificial sea salt 10 g in 1.0 L tap distilled H_2_O, pH 7.4–7.8) at 25 °C on a rotary platform shaker at 180 rpm for 72 h. Subsequently, a large-scale fermentation of the strain GXIMD 02518 was carried out in modified rice solid medium (100 g rice, 0.3 g corn steep liquor, 3 g artificial sea salt, and 150 mL H_2_O) employing 1 L × 100 Erlenmeyer flasks at room temperature for 30 days. The whole fermented cultures were extracted with EtOAc three times to provide a brown extract (168 g).

### 3.3. Extraction and Isolation

The crude extract was fractionated by medium pressure liquid chromatography (MPLC) using a gradient solvent system of petroleum ether–CH_2_Cl_2,_ followed by CH_2_Cl_2_–MeOH, yielding 15 fractions (Fr.1–Fr.15). Fr.3 was subjected to an ODS column with step-gradient elution of CH_3_OH−H_2_O, resulting in 13 subfractions (Fr.3.1–Fr.3.13). Fr.3.9 was further purified by semipreparative HPLC (70% CH_3_CN–H_2_O, 2 mL/min) to afford compounds **8** (5.0 mg, *t*_R_ 18 min) and **5** (6.6 mg, *t*_R_ 24 min). Meanwhile, Fr.7 was also chromatographed on a C_18_ ODS column, yielding 11 subfractions (Fr.7.1–Fr.7.11). Fr.7.9 was further purified by semipreparative HPLC (53% CH_3_CN–H_2_O, 2 mL/min) to give compounds **4** (3.3 mg, *t*_R_ 10.5 min), **6** (10.5 mg, tR 17.5 min), and **3** (4.4 mg, *t*_R_ 24 min). Fr.8 was processed on a C_18_ ODS column with step-gradient elution of CH_3_OH−H_2_O, affording 11 subfractions (Fr.8.1–Fr.8.11). Fr.8.2 was separated by semipreparative HPLC (27% CH_3_CN–H_2_O, 2 mL/min) to yield compounds **12** (4.5 mg, *t*_R_ 14 min) and **10** (6.4 mg, *t*_R_ 20 min). Fr.8.4 was purified using semipreparative HPLC (48% CH_3_CN–H_2_O, 2 mL/min) to give compounds **9** (12.4 mg, *t*_R_ 16 min), **2** (2.7 mg, *t*_R_ 23 min), and **1** (10.2 mg, *t*_R_ 26 min). Finally, Fr.8.7 was separated by semipreparative HPLC (45% CH_3_CN–H_2_O, 2 mL/min) to afford compounds **11** (9.7 mg, *t*_R_ 28 min) and **7** (4.1 mg, *t*_R_ 51 min).

Compound **1** was isolated as a racemate. Compound **1** was subjected to chiral HPLC (Chiralomix SA, 250 × 4.6 mm, 5 μm) using 70% n-hexane/isopropanol as the mobile phase to yield **1a** (4.3 mg, *t*_R_ 6 min) and **1b** (4.1 mg, *t*_R_ 6.8 min).

Cnidimol I (**1**): brown solid; **1a** [*α*${]}_{D}^{25}$ +44.1 (c 0.1, MeOH); ECD (0.25 mg/mL, MeOH) *λ*_max_ (∆*ε*) 204 (−4.54), 225 (13.33), 240 (5.60), 254 (8.55) nm. **1b** [*α*${]}_{D}^{25}$ −45.0 (c 0.1, MeOH); ECD (0.25 mg/mL, MeOH) *λ*_max_ (∆*ε*) 204 (5.82), 227 (−14.37), 242 (−6.08), 252 (−8.87) nm. UV (MeOH) *λ*_max_ (∆*ε*) 200 (3.20), 240 (3.25), 338 (2.69) nm; ^1^H NMR and ^13^C NMR (CDCl_3_) data, see Table 1; HRESIMS *m*/*z* 299.0537 [M + Na]^+^ (calcd. for C_14_H_12_O_6_Na, 299.0532).

Cnidimol J (**2**): brown solid; UV (MeOH) *λ*_max_ (∆*ε*) 227 (2.95), 242 (2.97), 344 (2.42) nm; ^1^H NMR and ^13^C NMR (CD_3_OD) data, see Table 1; HRESIMS *m*/*z* 299.0531 [M + Na]^+^ (calcd. for C_14_H_12_O_6_Na, 299.0532).

### 3.4. ECD Calculation of Compound ***1***

The ECD calculation was performed by the Gaussian 16 software, according to our previously reported method [9,10,29,30]. Briefly, conformational analysis was carried out using the Merck Molecular Force Field (MMFF) via Spartan’14 software (Wavefunction Inc., Irvine, CA, USA). The low-energy conformers with a Boltzmann population over 1% were subsequently subjected to DFT/TD-DFT calculations at the B3LYP/6−31+G (d) level in methanol, considering 50 excited states. Finally, the ECD spectrum was generated using SpecDis 1.71 (University of Wurzburg, Wurzburg, Germany).

### 3.5. Crystallographic Data for Compound ***1***

Compound **1** was crystallized from slow evaporation in CH_3_OH solution. A single crystal with the indicated dimensions was selected and measured on an XtalLAB PRO single-crystal diffractometer using Cu Kα radiation and refined by full-matrix least-squares calculation.

Molecular formula C_14_H_12_O_6_ (M = 276.24 g/mol): monoclinic, space group P2_1_/c, a = 11.8086(2) Å, b = 15.7680(2) Å, c = 7.04110(10) Å, β = 104.734(2), V = 1267.93(3) Å^3^, Z = 4, T = 99.98(11) K, μ(Cu Kα) = 0.975 mm^−1^, Dcalc = 1.447 g/cm^3^, 6300 reflections measured (7.742° ≤ 2Θ ≤ 147.516°), 2478 unique (R_int_ = 0.0253, R_sigma_ = 0.0307) which were used in all calculations. The final *R*_1_ was 0.0346 (I > 2σ(I)) and w*R*_2_ was 0.0956 (all data). The goodness of fit on F^2^ was 1.025. The crystallographic data for structure **1** had been deposited with the Cambridge Crystallographic Data Centre as supplementary publication numbers CCDC-2493886.

### 3.6. Anti-Osteoclastogenic Assay

All compounds were evaluated for their inhibitory activities of LPS-induced NF-κB activation in RAW264.7 cells, as detected by a luciferase reporter gene assay as described previously [9,10,11,12,13,14]. In brief, the RAW264.7 cells stably transfected with a luciferase reporter gene were plated in 96-well plates, and then pretreated with tested compounds (20 μM) and BAY11-7082 (NF-κB inhibitor as positive control, 5 μM, Sigma-Aldrich, Shanghai, China) for 30 min, followed by 5 μg/mL LPS stimulation for 8 h. Cells were harvested, and luciferase activities of the triplicate tests were measured by the luciferase assay system (Promega, Madison, WI, USA). To further explore the potential inhibitory effect of compound **6** on osteoclastogenesis, compound **6** (10, 15, and 20 μM) was added to bone marrow macrophage cells (BMMs, extracted from the femurs of C57BL/6 mice) with macrophage-stimulating factor (M-CSF) (50 ng/mL) and RANKL (100 ng/mL) stimulation for 3 days. Then the cells were fixed and stained to detect tartrate-resistant acidic phosphatase activity (TRAP), and the images were photographed using an inverted microscope (Nikon, Tokyo, Japan). The CCK-8 kit (Sigma-Aldrich) was used to evaluate the cytotoxic effects of **6** on BMMs as described previously.

### 3.7. Molecular Docking

The crystal structures of the NF-κB p65 protein (PDB ID: 3GUT) were retrieved from the Protein Data Bank (http://www.rcsb.org (accessed on 10 September 2025)) for molecular docking research, which was conducted by AutoDock (version 1.5.6). The structure of **6** was generated in ChemBio Office (version 17.0), followed by an MM2 calculation to minimize the conformation energy. The original ligand and crystal water were removed before the docking calculation. The hydrogens were added to the structure of NF-κB p65 protein, and Kollman united partial charges were assigned. A Lamarckian genetic algorithm was applied as a default search algorithm and allowed full flexibility of the active pockets of the ligand structure within the grid boxes size of 14 Å × 14 Å × 14 Å (x = 28.46, y = 93.55, z = 32.88). During the docking, the default parameters were used if it was not mentioned. The docking pose that had the lowest binding energy was represented as the most favorable binding conformation.

## 4. Conclusions

In summary, chemical investigation of the Beibu Gulf algicolous fungus *Aspergillus versicolor* GXIMD 02518 led to the isolation of two new chromone derivatives, cnidimols I and J (**1** and **2**), and ten known aromatic derivatives (**3**–**12**). Among them, compound **1** was a new pair of enantiomers. Its absolute configurations were determined using chiral-phase HPLC analysis, ECD calculations, and X-ray diffraction analysis. Compounds **6**–**8** exhibited inhibition of LPS-induced NF-κB activation in RAW 264.7 macrophages at 20 µM. Moreover, compound **6** further suppressed RANKL-induced osteoclast differentiation without any evidence of cytotoxicity in BMMs. Thus, this study provides a marine-derived drug lead for the treatment of osteoporosis.

## Figures and Tables

**Table 1:** ^1^H (500 MHz) and ^13^C NMR (125 MHz) data of compounds **1** (CDCl_3_) and **2** (CD_3_OD).

Position	1		2	
	*δ*_C_, Type	*δ*_H_, (*J* in Hz)	*δ*_C_, Type	*δ*_H_, (*J* in Hz)
2	169.4, C		171.8, C	
3	109.8, CH	6.23, s	110.6, CH	6.34, s
4	183.9, C		184.6, C	
4a	113.4, C		113.0, C	
5	156.5, C		159.2, C	
5a	126.2, C		126.1, C	
6	109.3, C		203.1, C	
7	166.7, C		167.4, C	
7a	133.8, C		136.6, C	
8	103.9, CH	7.36, s	109.8, CH	7.42, s
8a	158.3, C		158.1, C	
9	20.9, CH_3_	2.46, s	20.5, CH_3_	2.48, s
10	23.8, CH_3_	1.94, s	31.7, CH_3_	2.61, s
11	51.8, CH_3_	3.17, s	53.5, CH_3_	3.90, s

## Data Availability

The original data presented in the study are included in the article/Supplementary Materials; further inquiries can be directed to the corresponding author.

## Supplementary Materials

The following supporting information can be downloaded at: https://www.mdpi.com/article/10.3390/md23110429/s1: The ^1^H NMR spectrum of compound **1** in CDCl_3_ (500 MHz) (Figure S1), The ^13^C NMR spectrum of compound **1** in CDCl_3_ (125 MHz) (Figure S2), The HSQC spectrum of compound **1** in CDCl_3_ (Figure S3), The HMBC spectrum of compound **1** in CDCl_3_ (Figure S4), The HRESIMS spectrum of compound **1** in CH_3_OH (Figure S5), The UV spectrum of compound **1** in CH_3_OH (Figure S6), The 1H NMR spectrum of compound **2** in CD_3_OD (500 MHz) (Figure S7), The 13C NMR spectrum of compound **2** in CD_3_OD (125 MHz) (Figure S8), The HSQC spectrum of compound **2** in CD_3_OD (Figure S9), The HMBC spectrum of compound **2** in CD_3_OD (Figure S10), The HRESIMS spectrum of compound **2** in CH_3_OH (Figure S11), The UV spectrum of compound **2** in CH_3_OH (Figure S12), chiral separation chromatograms of enantiomers **1a**/**1b** (Figure S13), the optimized conformers and equilibrium populations of **1** (Figure S14). Table S1. Energies of **1** at MMFF94 force field, Table S2. Energies of **1** at B3LYP/6–31+g(d) level in methanol.
