# Supplementary material for: Two New Chromone Derivatives from a Marine Algicolous Fungus Aspergillus versicolor GXIMD 02518 and Their Osteoclastogenesis Inhibitory Activity
Source: Mar Drugs. 2025 Nov 7;23(11):429. doi: 10.3390/md23110429 (PMC12653716; doi:10.3390/md23110429)
Supplement: Supplementary file 1 [file marinedrugs-23-00429-s001.zip › marinedrugs-3949929-supplementary.pdf]

Two new chromone derivatives from a marine algicolous fungus *Aspergillus versicolor* GXIMD 02518 and their osteoclastogenesis inhibitory activity

Xin Qi[1,†], Zhen Li [2,†], Miaoping Lin[1], Humu Lu[1], Shuai Peng[1], Huangxue Qin [1], Yonghong Liu[1], Chenghai Gao[1,*], Xiaowei Luo[1,*]

[1] Guangxi Key Laboratory of Marine Drugs, University Engineering Research Center of High-efficient Utilization of Marine Traditional Chinese Medicine Resources, Guangxi, Institute of Marine Drugs, Guangxi University of Chinese Medicine, Nanning 530200, China;

[2] School of Basic Medical Sciences, Hunan University of Medicine, Huaihua 418000, China.;

* Correspondence: luoxiaowei1991@126.com (X.L.); gaoch@gxtcmu.edu.cn (C.G.);

† These authors contributed equally to this work.

List of Supporting Information

The ITS gene sequence data of *Aspergillus versicolor* GXIMD 02518. 3

Phylogenetic tree of *Aspergillus versicolor* GXIMD 02518...................................... 3

The physicochemical data of compounds 3–12. 3

Figure S1. The ^{1}H NMR spectrum of compound **1** in $CDCl_3$ (500 MHz). 6

Figure S2. The ^{13}C NMR spectrum of compound **1** in $CDCl_3$ (125 MHz). 6

Figure S3. The HSQC spectrum of compound **1** in $CDCl_3$... 7

Figure S4. The HMBC spectrum of compound **1** in $CDCl_3$. 7

Figure S5. The HRESIMS spectrum of compound **1** in CH_3OH. 8

Figure S6. The UV spectrum of compound **1** in CH_3OH... 8

Figure S7. The 1H NMR spectrum of compound **2** in CD_3OD (500 MHz). 9

Figure S8. The 13C NMR spectrum of compound **2** in CD_3OD (125 MHz)............... 9

Figure S9. The HSQC spectrum of compound **2** in CD_3OD...................................... 10

Figure S10. The HMBC spectrum of compound **2** in CD_3OD. 10

Figure S11. The HRESIMS spectrum of compound **2** in CH_3OH. 11

Figure S12. The UV spectrum of compound **2** in CH_3OH... 11

Figure S13. Chiral separation chromatograms of enantiomers **1a**/**1b**. 12

Table S1. Energies of **1** at MMFF94 force field... 12

Table S2. Energies of **1** at B3LYP/6–31+g(d) level in methanol. 12

Figure S14. The optimized conformers and equilibrium populations of **1**. 13

The ITS gene sequence data of *Aspergillus versicolor* GXIMD 02518.

CTCCCACCCGTGACTACCTAACACTGTTGCTTCGGCGGGGAGCCCTCTCGG
GGGCGCGCCGCCGGGGACTACTGAACTTCATGCCTGAGAGTGATGCAGTC
TGAGTCTGAATATAAAATCAGTCAAAACTTTCAACAATGGATCTCTTGGTTC
CGGCATCGATGAAGAACGCAGCGAACTGCGATAAGTAATGTGAATTGCAG
AATTCAGTGAATCATCGAGTCTTTGAACGCACATTGCGCCCCCTGGCATTCC
GGGGGGCATGCCTGTCCGAGCGTCATTGCTGCCCATCAAGCCCGGCTTGTG
TGTTGGGTCGTCGTCCCCCCCGGGGGACGGGCCCGAAAGGCAGCGGCGGC
ACCGTGTCCGGTCCTCGAGCGTATGGGGCTTTGTCACCCGCTCGATTTAGG
GCCGGCCGGGCGCCAGCCGACGTCCAACCATTTTTCTTCAGGTTGACCTCG
GATCAGGTAGGGATACCCGCTGAACTTAAGCAATA

Phylogenetic tree of *Aspergillus versicolor* GXIMD 02518.

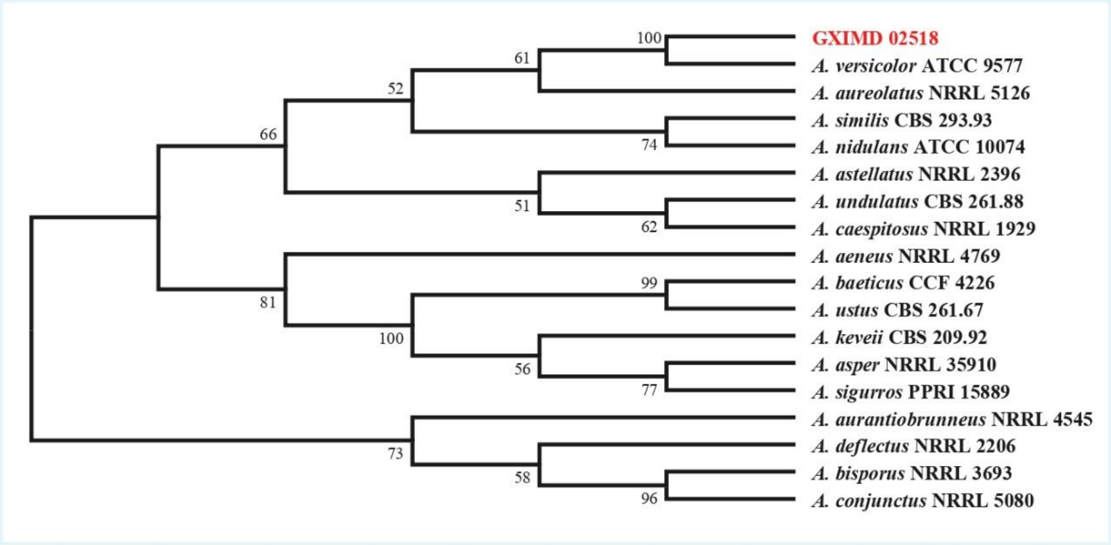

The physicochemical data of compounds 3–12.

diorcinol (**3**): red solid; ^{1}H NMR (500 MHz, CD_3OD), δ_H: 6.35 (2H, s, H-6, 6′), 6.25 (2H, s, H-4, 4′), 6.19 (2H, s, H-2, 2′), 2.21 (6H, s, H-7, 7′); ^{13}C NMR (125 MHz, CD_3OD), δ_C: 159.7 (C, C-1, 1′), 159.4 (C, C-3, 3′), 141.6 (C, C-5, 5′), 112.0 (CH, C-4, 4′), 111,7 (CH, C-6, 6′), 104.3 (CH, C-2, 2′), 21.5 (CH_3, C-7, 7′).

diorcinol-3-*O*-*α*-D-ribofuranoside (**4**): red solid; ^{1}H NMR (500 MHz, CD_3OD), δ_H:

6.73 (1H, s, H-6′), 6.57 (1H, s, H-2′), 6.46 (1H, s, H-4′), 6.37 (1H, s, H-6), 6.28 (1H, s, H-4), 6.20 (1H, s, H-2), 5.58 (1H, d, J = 4.5 Hz, H-1″), 4.16 (1H, dd, J = 6.5, 4.5 Hz, H-2″), 4.12 (1H, dd, J = 6.9, 3.5 Hz, H-4″), 4.07 (1H, dd, J = 6.5, 3.1 Hz, H-3″), 3.70 (1H, dd, J = 12.1, 3.4 Hz, H-5″a), 3.64 (1H, dd, J = 12.2, 4.0 Hz, H-5″b), 2.28 (3H, s, H-7), 2.23 (3H, s, H-7′); ^{13}C NMR (125 MHz, CD_3OD), δ_C: 159.8 (C, C-1′), 159.6 (C, C-1), 159.5 (C, C-3), 159.4 (C, C-3′), 141.7 (C, C-5′), 141.6 (C, C-5), 114.2 (CH, C-4′), 113.5 (C, C-1′), 112.1(CH, C-6), 111.8 (CH, C-4), 106.3 (CH, C-2), 104.3 (CH, C-2′), 102.3 (CH, C-1″), 87.5 (CH, C-4″), 73.4 (CH, C-2″), 71.2 (CH, C-3″), 63.2 (CH_2, C-5″), 21.6 (CH_3, C-7), 21.5 (CH_3, C-7′).

methyl 2-hydroxy-4-(3-hydroxy-5-methylphenoxy)-6-methylbenzoate (**5**): brown oil; ^{1}H NMR (500 MHz, CD_3OD), δ_H: 6.44 (1H, s, H-14), 6.30 (2H, s, H-4, 12), 6.24 (1H, s, H-10), 6.18 (1H, s, H-6), 3.88 (3H, s, 1-OCH_3), 2.41 (3H, s, H-8), 2.22 (3H, s, H-15); ^{13}C NMR (125 MHz, CD_3OD), δ_C: 172.9 (C, C-1), 164.9 (C, C-7), 163.6 (C, C-5), 159.9 (C, C-11), 157.3 (C, C-9), 144.2 (C, C-3), 142.2 (C, C-13), 113.6 (CH, C-14), 113.3 (CH, C-4), 113.1 (CH, C-12), 109.2 (C, C-2), 105.7 (CH, C-10), 103.6 (CH, C-6), 52.4 (CH_3, 1-OCH_3), 23.8 (CH_3, C-8), 21.5 (CH_3, C-15).

3,7-dihydroxy-1,9-dimethyldibenzofuran (**6**): red solid; ^{1}H NMR (500 MHz, CD_3OD), δ_H: 6.66 (2H, s, H-4, 6), 6.51 (2H, s, H-2, 8), 2.73 (6H, s, 1-Me, 9-Me); ^{13}C NMR (125 MHz, CD_3OD), δ_C: 159.1 (C, C-4a, 5a), 156.9 (C, C-3, 7), 132.9 (C, C-1, 9), 117.6 (C, C-9a, 9b), 114.8 (CH, C-2, 8), 96.5 (CH, C-4, 6), 25.0 (CH_3, 1-Me, 9-Me).

monomethylsulochrin (**7**): white solid; ^{1}H NMR (500 MHz, DMSO-d_6), δ_H: 6.89 (1H, d, J = 2.1 Hz, H-5), 6.69 (1H, d, J = 2.1 Hz, H-3), 6.38 (1H, s, H-5′), 6.26 (1H, s, H-3′), 3.63 (3H, s, H-9), 3.62 (3H, s, H-8), 3.33 (3H, s, H-7′), 2.26 (3H, s, H-8′); ^{13}C NMR (125 MHz, DMSO-d_6), δ_C: 125.8 (C, C-1), 156.7 (C, C-2), 103.2 (CH, C-3), 158.2 (C, C-4), 107.2 (CH, C-5), 128.0 (C, C-6), 165.8 (C, C-7), 52.2 (CH_3, C-8), 56.0 (CH_3, C-9), 199.4 (C, C-10), 110.1 (C, C-1′), 160.8 (C, C-2′), 103.5 (CH, C-3′), 147.9 (C, C-4′), 110.1 (CH, C-5′), 163.3 (C, C-6′), 56.0 (CH_3, C-7′), 22.0 (CH_3, C-8′).

sterigmatocystin (**8**): white solid; ^{1}H NMR (500 MHz, DMSO-d_6), δ_H: 6.74 (2H, m, H-4, 17), 7.62 (1H, t, J = 8.3 Hz, H-5), 6.98 (2H, m, H-6, 14), 6.71 (1H, s, H-11), 4.84 (1H, m, H-15), 5.53 (1H, t, J = 2.6 Hz, H-16), 3.88 (3H, s, H-18); ^{13}C NMR (125

MHz, DMSO-d_6), δ_C: 180.4 (C, C-1), 108.2 (C, C-2), 161.4 (C, C-3), 110.8 (CH, C-4), 136.2 (CH, C-5), 106.5 (CH, C-6), 154.5 (C, C-7), 153.3 (C, C-8), 106.5 (C, C-9), 164.4 (C, C-10), 91.0 (CH, C-11), 162.9 (C, C-12), 104.9 (C, C-13), 113.4 (CH, C-14), 47.3 (CH, C-15), 102.6 (C, C-16), 145.6 (CH, C-17), 56.8 (CH_3, C-18).

aflaquinolone E (**9**): white solid; ^{1}H NMR (500 MHz, CD_3OD), δ_H: 3.67 (1H, s, H-3), 6.47 (1H, d, *J* = 8.0 Hz, H-7), 7.16 (1H, t, *J* = 8.1 Hz, H-8), 6.55 (1H, d, *J* = 8.3 Hz, H-9), 7.30 (5H, m, H-12, 13, 14, 15, 16), 3.54 (3H, s, H-17); ^{13}C NMR (125 MHz, CD_3OD), δ_C: 169.1 (C, C-2), 86.3 (CH, C-3), 79.9 (C, C-4), 113.0 (C, C-5), 159.1 (C, C-6), 108.2 (CH, C-7), 131.0 (CH, C-8), 113.2 (CH, C-9), 138.1 (C, C-10), 140.9 (C, C-11), 127.5 (CH, C-12, 16), 129.6 (CH, C-13, 15), 129.8 (CH, C-14), 59.2 (CH_3, C-17).

4-(hydroxy(4-hydroxyphenyl) methoxy) benzaldehyde (**10**): white solid; ^{1}H NMR (500 MHz, CD_3OD), δ_H: 9.78 (1H, s, H-1), 7.79 (2H, d, *J* = 8.6 Hz, H-3, 7), 6.93 (2H, d, *J* = 8.6 Hz, H-4, 6), 5.28 (1H, s, H-1′), 7.24 (2H, d, *J* = 8.5 Hz, H-3′, 7′), 6.77 (2H, d, *J* = 8.6 Hz, H-4′, 6′); ^{13}C NMR (125 MHz, CD_3OD), δ_C: 192.8 (C, C-1), 130.3 (C, C-2), 133.4 (CH, C-3, 7), 115.8 (CH, C-4, 6), 165.1 (C, C-5), 104.8 (CH, C-1′), 130.4 (C, C-2′), 129.0 (CH, C-3′, 7′), 116.8 (CH, C-4′, 6′), 158.8 (C, C-5′).

aspergoterpenin D (**11**): yellow oil; ^{1}H NMR (500 MHz, CD_3OD), δ_H: 7.25 (1H, dd, *J* = 8.1, 1.4 Hz, H-3), 7.41 (1H, dd, *J* = 8.1, 1.8 Hz, H-4), 7.33 (1H, s, H-6), 1.93 (1H, m, H-9a), 1.75 (1H, m, H-9b), 1.28 (2H, m, H-10), 1.37 (1H, m, H-11a), 1.09 (1H, m, H-11a), 1.68 (1H, m, H-12), 0.81 (3H, d, *J* = 6.7 Hz, H-17), 3.83 (1H, m, H-14a), 3.75 (1H, m, H-14b), 1.56 (3H, s, H-15), 1.95 (3H, s, H-17); ^{13}C NMR (125 MHz, CD_3OD), δ_C: 156.8 (C, C-1), 137.9 (C, C-2), 127.8 (CH, C-3), 121.5 (CH, C-4), 131.6 (C, C-5), 118.6 (CH, C-6), 169.9 (C, C-7), 77.7 (C, C-8), 43.4 (CH_2, C-9), 22.4 (CH_2, C-10), 34.8 (CH_2, C-11), 33.6 (CH, C-12), 17.1 (CH_3, C-13), 70.5 (CH_2, C-14), 28.9 (CH_3, C-15), 173.1 (C, C-16), 20.8 (CH_3, C-17).

vanillic acid (**12**): white solid; ^{1}H NMR (500 MHz, CD_3OD), δ_H: 7.56 (1H, m, H-2), 6.85 (1H, d, *J* = 8.7 Hz, H-5), 7.56 (1H, m, H-6), 3.90 (3H, s, 3-OCH_3); ^{13}C NMR (125 MHz, CD_3OD), δ_C: 123.0 (C, C-1), 115.8 (CH, C-2), 148.7 (C, C-3), 152.7 (C,

C-4), 113.8 (CH, C-5), 125.3 (CH, C-6), 170.0 (C, 1-COOH), 56.4 (CH_3, 3-OCH_3).

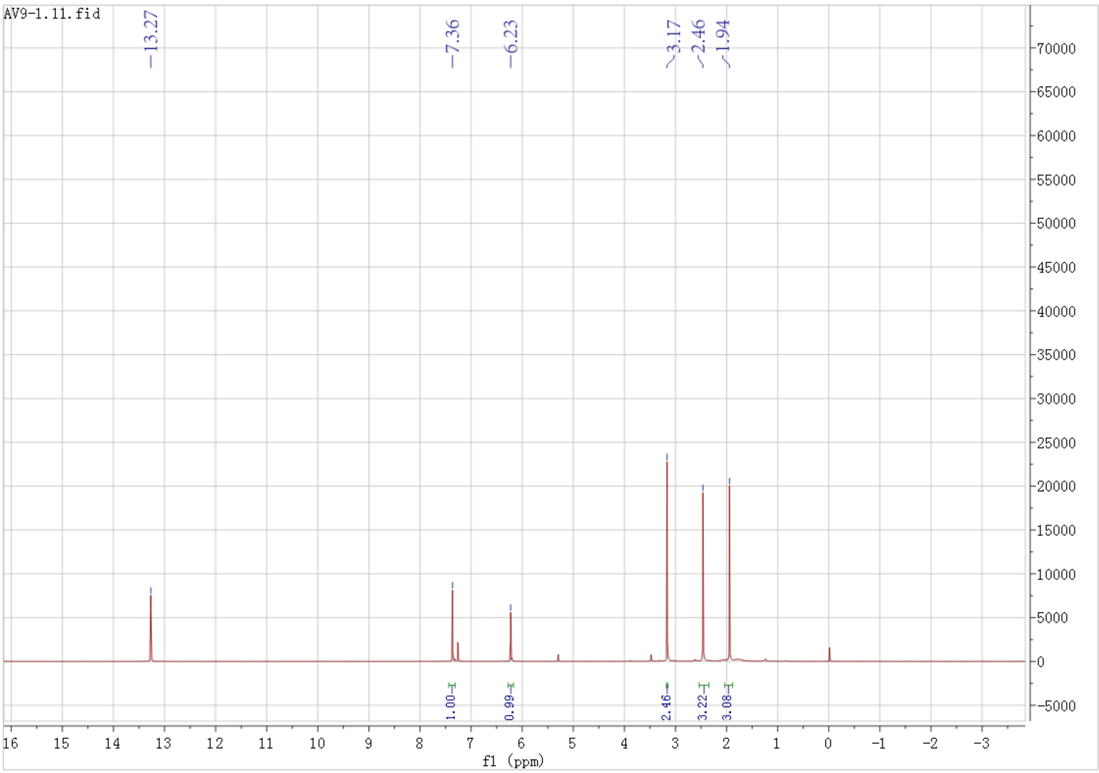

Figure S1. The ^{1}H NMR spectrum of compound **1** in $CDCl_3$ (500 MHz).

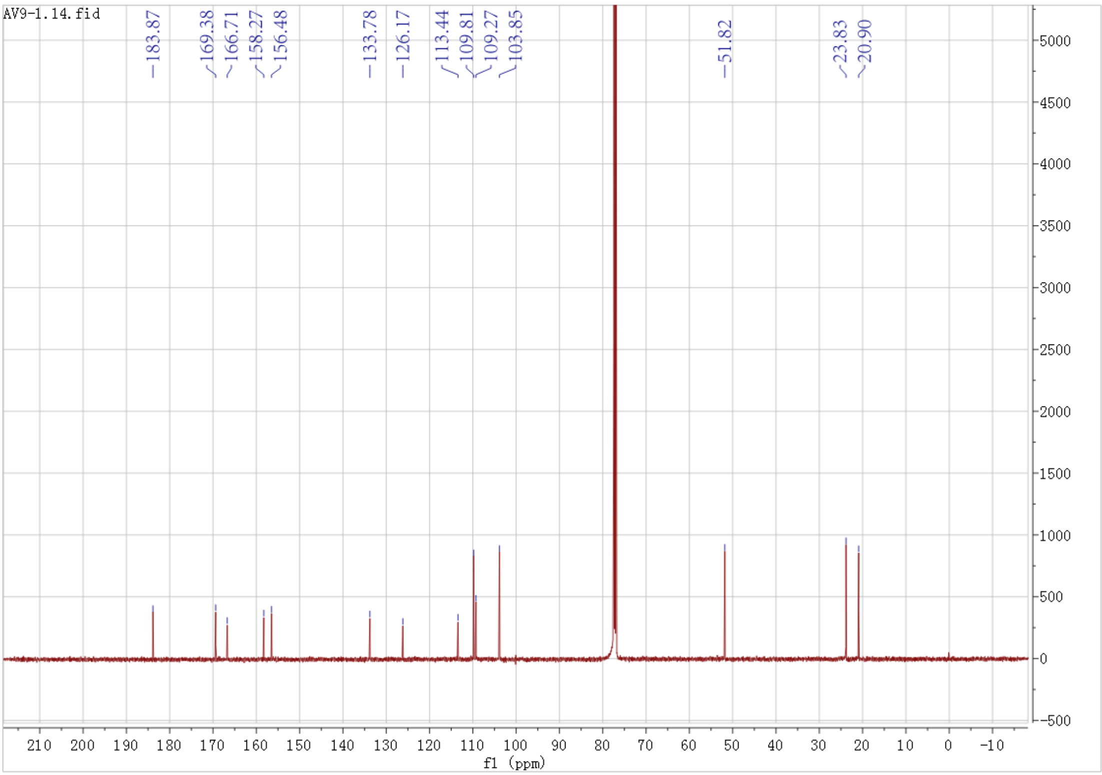

Figure S2. The ^{13}C NMR spectrum of compound **1** in $CDCl_3$ (125 MHz).

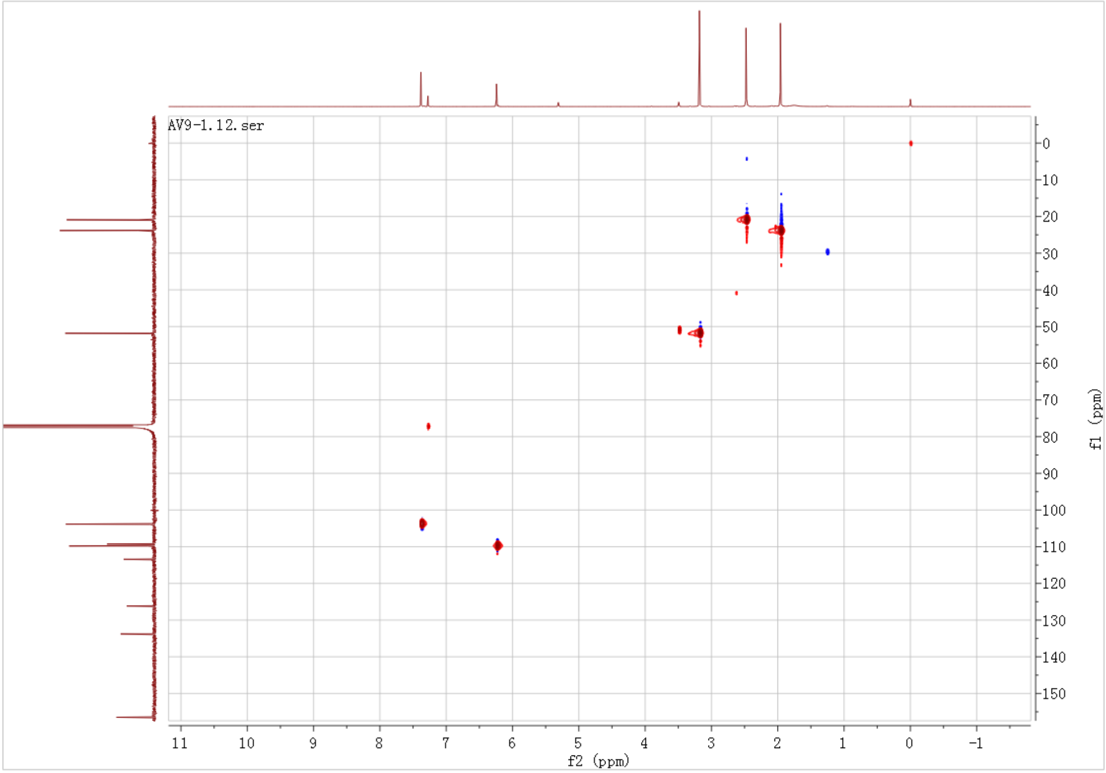

Figure S3. The HSQC spectrum of compound **1** in $CDCl_3$.

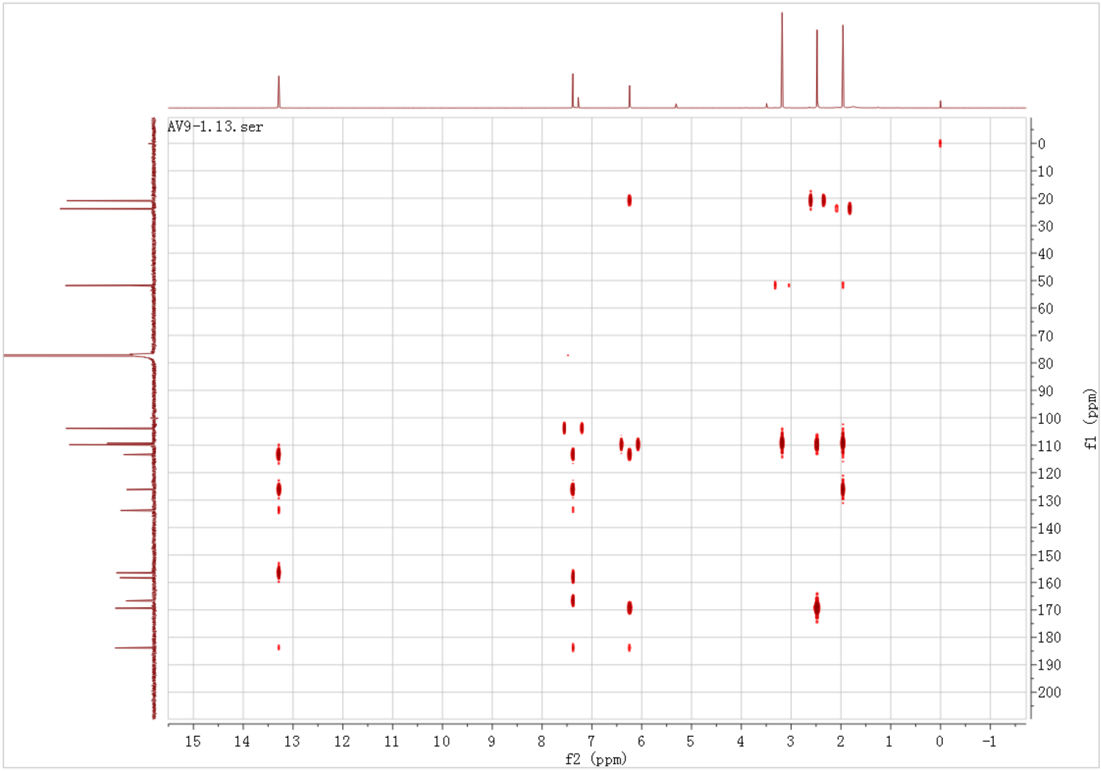

Figure S4. The HMBC spectrum of compound **1** in $CDCl_3$.

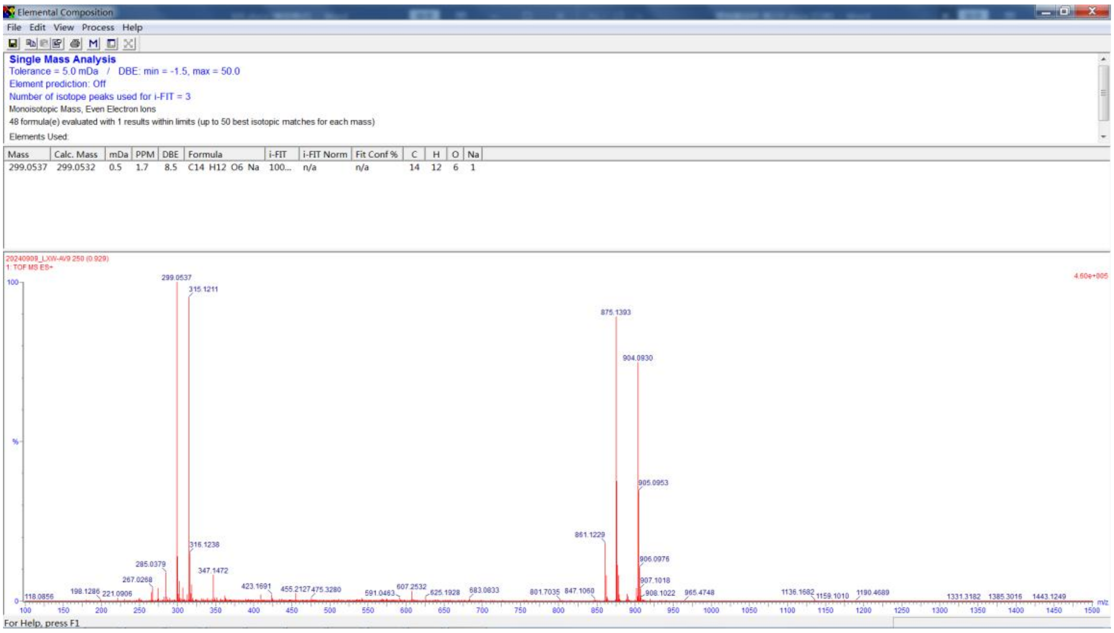

Figure S5. The HRESIMS spectrum of compound **1** in CH_3OH.

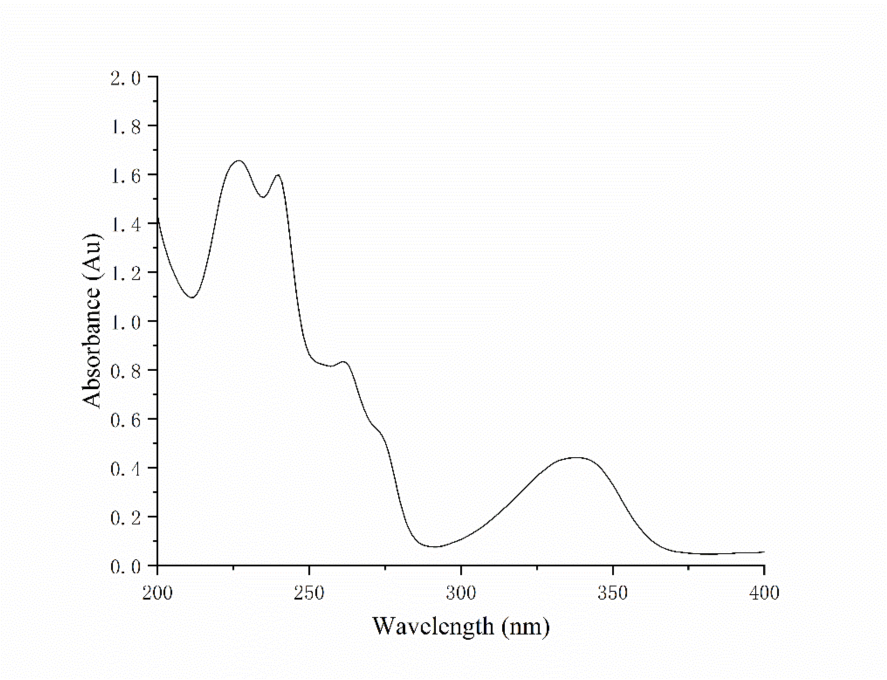

Figure S6. The UV spectrum of compound **1** in CH_3OH.

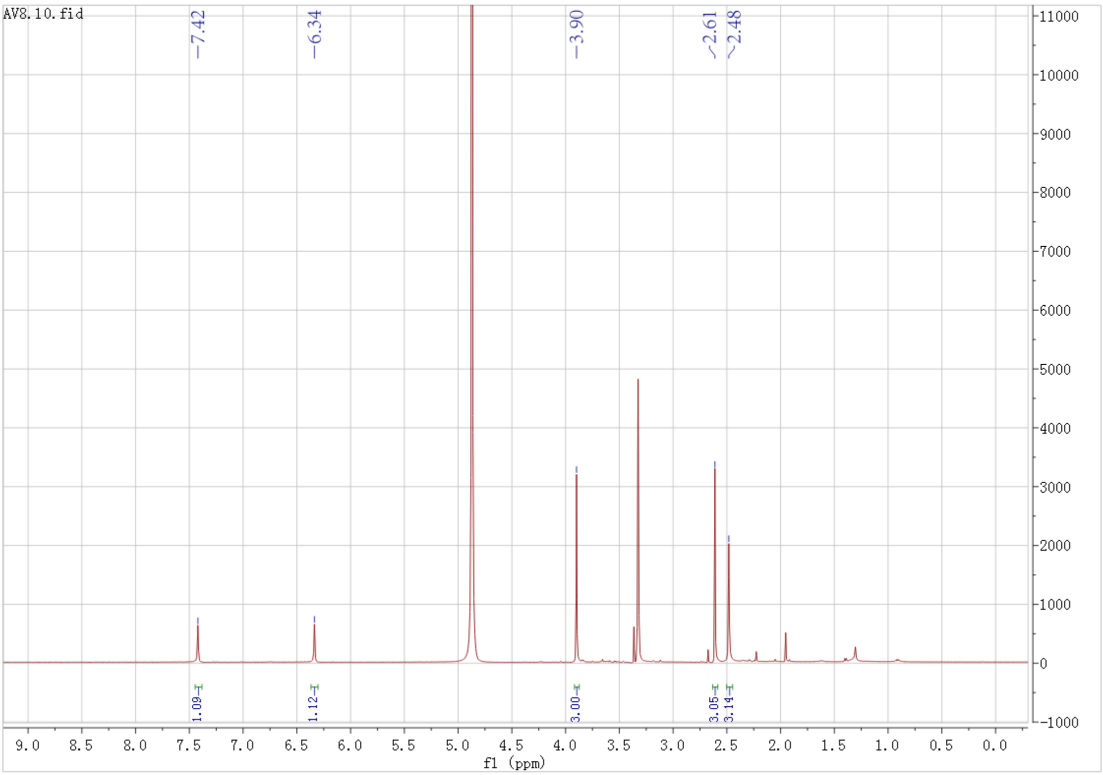

Figure S7. The ^{1}H NMR spectrum of compound **2** in CD_3OD (500 MHz).

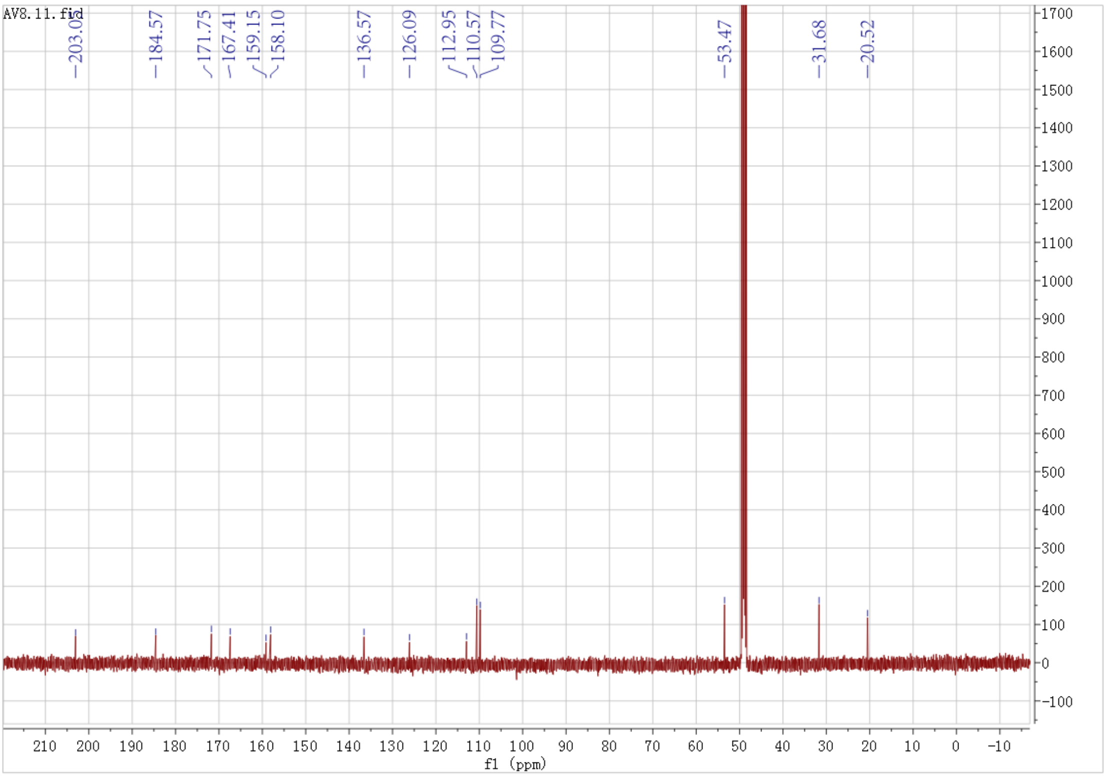

Figure S8. The ^{13}C NMR spectrum of compound **2** in CD_3OD (125 MHz).

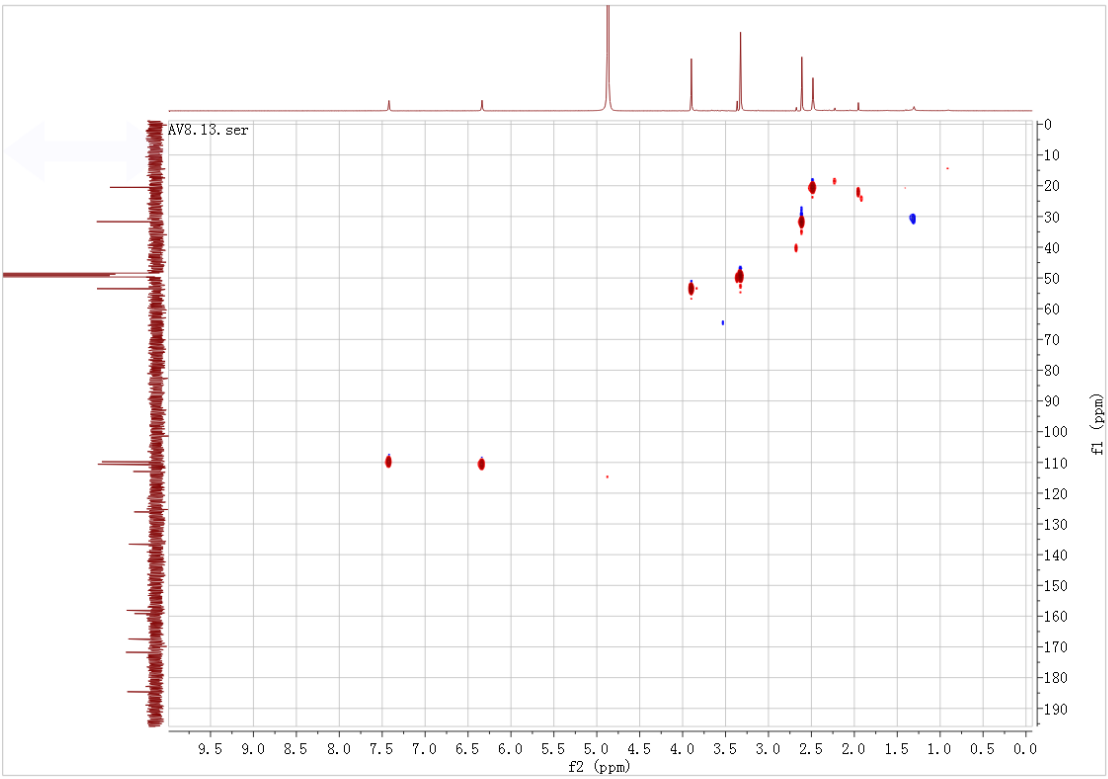

Figure S9. The HSQC spectrum of compound **2** in CD_3OD.

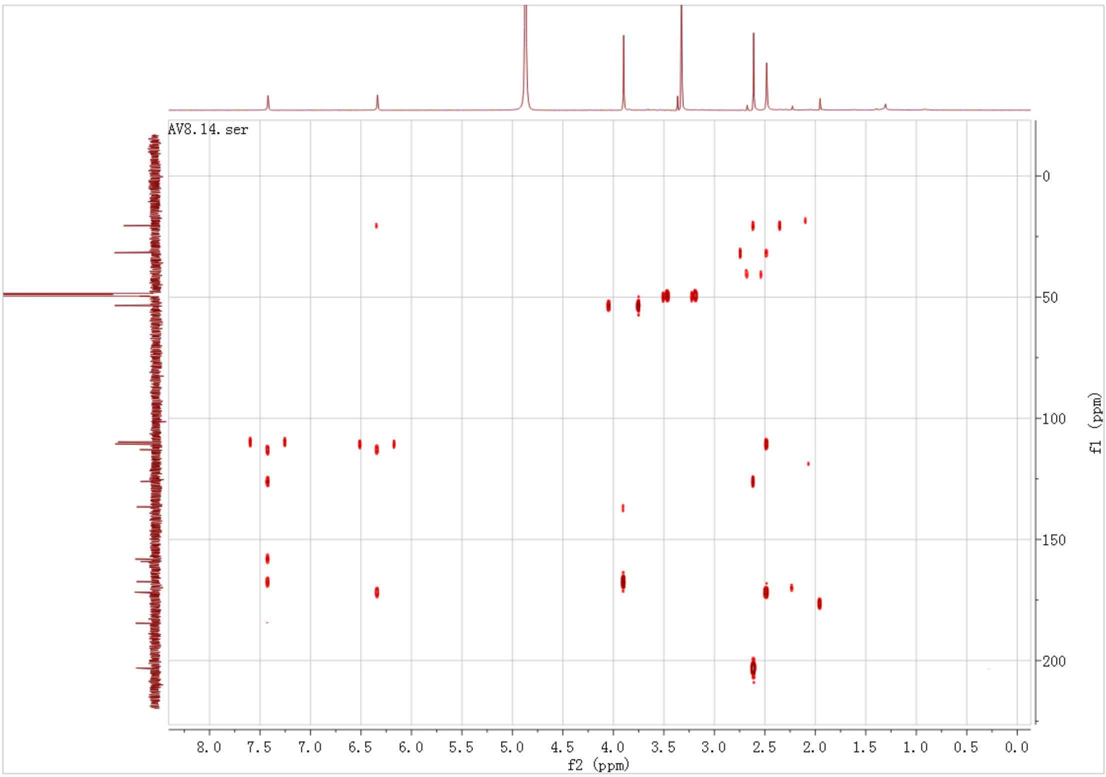

Figure S10. The HMBC spectrum of compound **2** in CD_3OD.

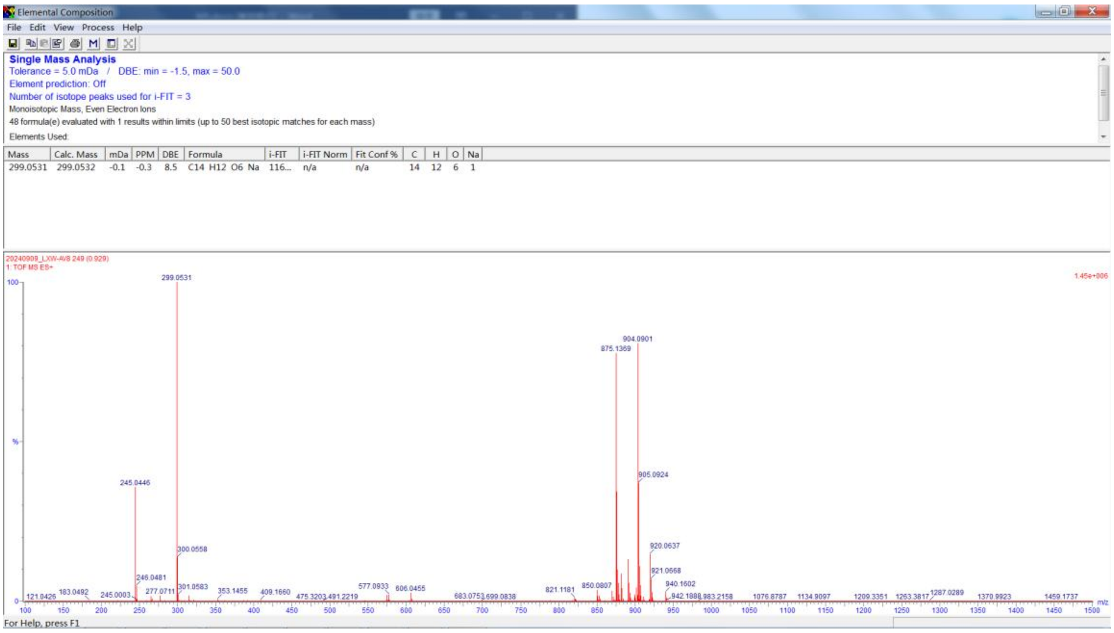

Figure S11. The HRESIMS spectrum of compound **2** in CH_3OH.

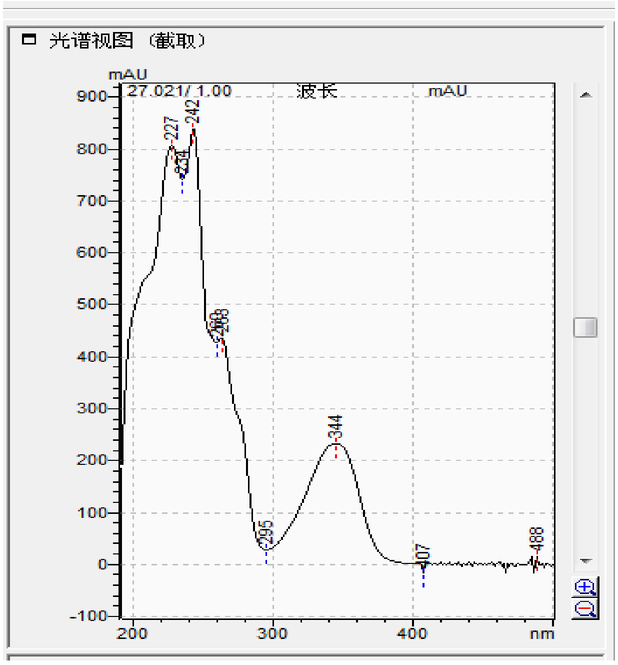

Figure S12. The UV spectrum of compound **2** in CH_3OH.

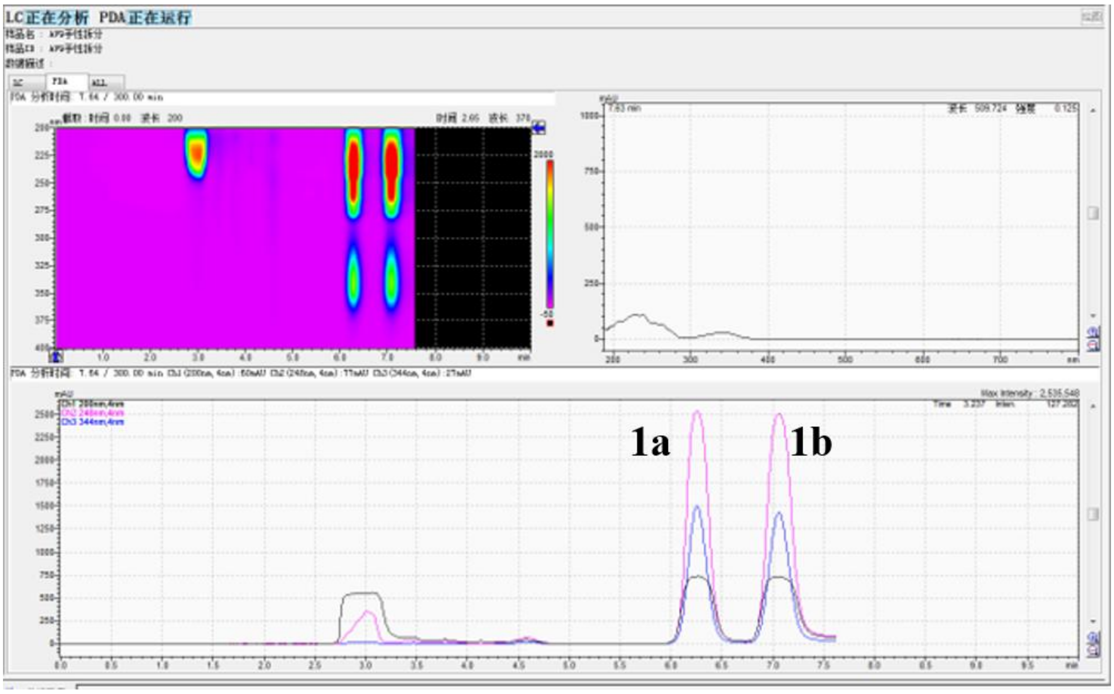

Figure S13. Chiral separation chromatograms of enantiomers **1a**/**1b**.

Table S1. Energies of **1** at MMFF94 force field.

Configuration	Conformer	Energy (kcal/mol)	Population (%)
R-**1**	1	16.35	93.5
R-**1**	2	17.98	6.0
R-**1**	3	19.48	0.5
S-**1**	1	16.35	93.5
S-**1**	2	17.98	6.0
S-**1**	3	19.48	0.5

Table S2. Energies of **1** at B3LYP/6–31+g(d) level in methanol.

Configuration	Conformer	E (Hartree)	Energy (kcal/mol)	Population (%)
R-**1**	1	−992.3753934	−622724.99	14.19
R-**1**	2	−992.3771227	−622726.07	85.81
S-**1**	1	−992.3753932	−622724.99	14.18
S-**1**	2	−992.3771228	−622726.07	85.75
S-**1**	3	−992.3702206	−622721.74	0.07

R-**1**

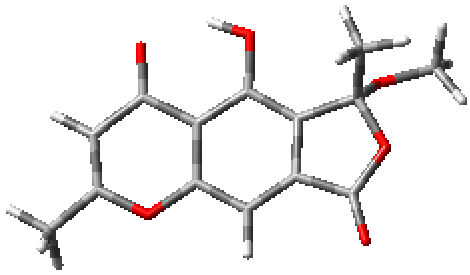

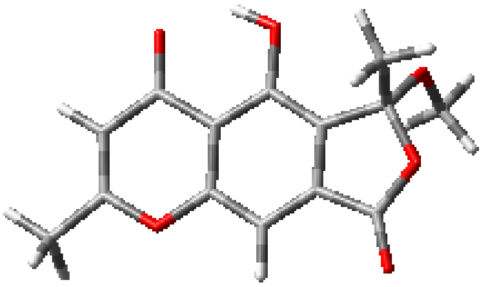

S-**1**

Conf.1 (14.19%)

Conf.2 (85.81%)

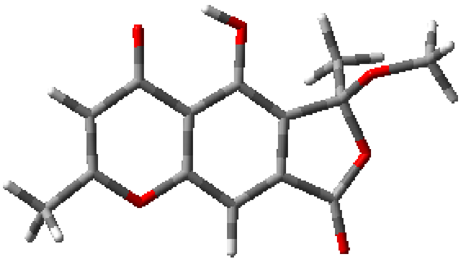

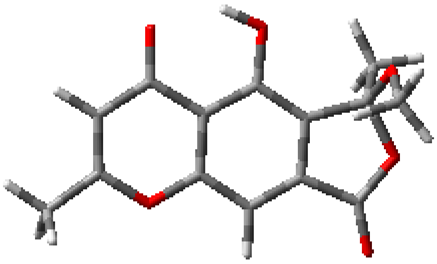

Conf.1 (14.18%)

Conf.2 (85.75%)

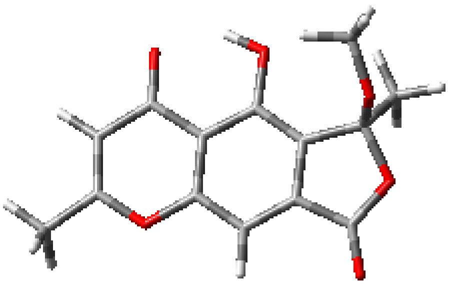

Conf.1 (0.07%)

Figure S14. The optimized conformers and equilibrium populations of **1**.
